# Dynamic micro-optical coherence tomography enables structural and metabolic imaging of the mammalian cochlea

**DOI:** 10.3389/fnmol.2024.1436837

**Published:** 2024-10-10

**Authors:** Hinnerk Schulz-Hildebrandt, Svetolik Spasic, Fang Hou, Kuan-Chung Ting, Shelley Batts, Guillermo Tearney, Konstantina M. Stankovic

**Affiliations:** ^1^Wellman Center for Photomedicine, Massachusetts General Hospital, Harvard Medical School, Boston, MA, United States; ^2^Department of Otolaryngology-Head and Neck Surgery, Stanford University School of Medicine, Stanford, CA, United States; ^3^Department of Pathology, Massachusetts General Hospital, Boston, MA, United States; ^4^Harvard-MIT Division of Health Science and Technology, Cambridge, MA, United States; ^5^Department of Neurosurgery, Stanford University School of Medicine, Stanford, CA, United States; ^6^Wu Tsai Neurosciences Institute, Stanford University, Stanford, CA, United States

**Keywords:** cochlea, hair cell, metabolic imaging, micro-optical coherence tomography, organ of Corti, sensorineural hearing loss

## Abstract

Sensorineural hearing loss (SNHL) is caused by damage to the mechanosensory hair cells and auditory neurons of the cochlea. The development of imaging tools that can directly visualize or provide functional information about a patient’s cochlear cells is critical to identify the pathobiological defect and determine the cells’ receptiveness to emerging SNHL treatments. However, the cochlea’s small size, embedded location within dense bone, and sensitivity to perturbation have historically precluded high-resolution clinical imaging. Previously, we developed micro-optical coherence tomography (μOCT) as a platform for otologic imaging in animal models and human cochleae. Here we report on advancing μOCT technology to obtain simultaneously acquired and co-localized images of cell viability/metabolic activity through dynamic μOCT (DμOCT) imaging of intracellular motion. DμOCT obtains cross-sectional images of ATP-dependent movement of intracellular organelles and cytoskeletal polymerization by acquiring sequential μOCT images and computing intensity fluctuation frequency metrics on a pixel-wise basis. Using a customized benchtop DμOCT system, we demonstrate the detailed resolution of anatomical and metabolic features of cells within the organ of Corti, via an apical cochleostomy, in freshly-excised adult mouse cochleae. Further, we show that DμOCT is capable of capturing rapid changes in cochlear cell metabolism following an ototoxic insult to induce cell death and actin stabilization. Notably, as few as 6 frames can be used to reconstruct cochlear DμOCT images with sufficient detail to discern individual cells and their metabolic state. Taken together, these results motivate future development of a DμOCT imaging probe for cellular and metabolic diagnosis of SNHL in humans.

## 1 Introduction

Sensorineural hearing loss (SNHL) is caused by the loss or dysfunction of cochlear hair cells and/or spiral ganglion neurons (SGNs) which, respectively, detect sound vibrations in the cochlea and transmit this information to the brain (Tanna et al., 2023). In mammals, these sensory cells are present at birth, are not replaced when lost, and have limited ability for repair (Chen et al., 2019). Thus, their damage due to aging (Bowl and Dawson, 2019), loud noise (Natarajan et al., 2023), genetic mutations (Bommakanti et al., 2019), or other factors (i.e., trauma, ototoxicity, inflammation, or infections) (van de Water et al., 2010; Ganesan et al., 2018) all contribute to SNHL being a leading sensory impairment globally (World Health Organization, 2024).

The presentation of SNHL greatly varies from patient to patient—the deficit may be mild or profound, present in one ear or both, or affect certain frequencies (high or low) or all (Schilder et al., 2017). SNHL may be gradual or sudden (Chandrasekhar et al., 2019) and can be present from birth or appear in later life (Punnoose et al., 2012; Korver et al., 2017). Although the cellular etiology underlying each patient’s SNHL is unique, current diagnostic methods primarily identify only the presence and degree of hearing loss. For most patients, the underlying cellular defect(s) remain unknown during clinical evaluation, selection into clinical trials, and assessment of therapeutic results (Le Prell, 2021). Instead, our knowledge of the cellular pathophysiology of SNHL is limited to studies using animal models and specimens from human cadavers or organ donors.

Unlike other medical disciplines, otolaryngology has not benefitted from the transformative impact of high-resolution clinical imaging which could help overcome our limited knowledge of living human anatomy and dynamics. This is primarily due to the small size, complex spiraling structure, and embedded location of the human cochlea. Cochlear sensorineural cells lie within in the hard temporal bone, precluding non-destructive biopsy to assess their status, and are 10–50 microns in size (Gao et al., 2011), placing them outside the resolution of conventional imaging like computed tomography or magnetic resonance imaging (Arnold et al., 1996; Lane et al., 2004; Lane et al., 2008; Hiremath et al., 2023). Clinical light microscopes and rigid endoscopes routinely used to visualize the middle ear during otologic surgery similarly cannot provide insight on inner ear sensorineural cell defects due to lack of access, resolution, contrast, and optical sectioning (Chole, 2015). Therefore, there is a need for an *in vivo* imaging tool that can determine the status of cochlear sensorineural cells, the underlying pathobiological defect, and the receptiveness of these cells to emerging SNHL treatments.

Optical coherence tomography (OCT) is a cross-sectional, natural contrast imaging technique that measures the intensity of backscattered light from microstructural features at varying depths within biological tissues (Huang et al., 1991; Tearney et al., 1997). OCT acquires high resolution (10–30 μm) cross-sectional images and has been used to assess cochlear morphology and mechanics in animal models *in vivo* and *ex vivo* (Wong et al., 2000; Lin et al., 2008; Subhash et al., 2010; Gao et al., 2011; Kahrs et al., 2011; Dong et al., 2018). Its capability to resolve the cochlea’s basilar membrane has enabled measurement of high-frequency mechanical vibrations in response to sound (Hong and Freeman, 2006; Subhash et al., 2010; Wang and Nuttall, 2010; Dong et al., 2018). The resolution of OCT is also sufficient for detecting other anatomical features like the boundaries between the cochlea’s fluid-filled compartments (Wong et al., 2000; Lin et al., 2008; Subhash et al., 2010), tectorial membrane (Lin et al., 2008; Subhash et al., 2010; Wang and Nuttall, 2010; Gao et al., 2011), tunnel of Corti (Gao et al., 2011), and sensory epithelium (Lin et al., 2008; Subhash et al., 2010; Gao et al., 2011).

OCT faces challenges in resolving individual cells or sub-cellular features due to its limited resolution that is compounded by speckle noise arising from coherent light detection (Schmitt et al., 1999). Furthermore, because OCT is fundamentally based on changes in the refractive index and scattering properties, it cannot assess cell viability and metabolic activity. For these reasons, studies using standard OCT used for imaging the inner ear (Gao et al., 2011; Starovoyt et al., 2019) demonstrated insufficient resolution for further clinical development. We overcame these limitations by developing μOCT, an advanced form of OCT with higher resolution of up to 1 μm (Liu et al., 2011). μOCT achieves superior axial resolution over conventional OCT by using a very broad bandwidth source and lateral resolution via higher numerical apertures (NA) and extended depth of focus (EDOF) optics (Liu et al., 2011; Iyer et al., 2016). Imaging extracted rodent cochlea revealed that μOCT can visualize individual outer hair cells (OHCs) and Hensen’s cells (Iyer et al., 2016), which are absent in noise-damaged tissue (Iyer et al., 2021). Additionally, 3D μOCT enabled visualization of discrete outer pillar cells, the tectorial, basilar, and Reissner’s membranes, and SGN fibers crossing the tunnel of Corti and space of Nuel to the OHCs, their synaptic partners (Iyer et al., 2016).

We recently reported further advancement of μOCT technology to overcome speckle noise and obtain information of cell viability/metabolic activity through dynamic μOCT (DμOCT) imaging of intracellular motion in freshly excised tissue (Leung et al., 2020). DμOCT obtains sequential μOCT cross-sectional images of ATP-dependent movement of intracellular organelles (e.g., vesicular transport, cytoskeletal polymerization) and computes intensity fluctuation frequency metrics on a pixel-wise basis (Leung et al., 2020; Münter et al., 2020). Here, we demonstrate that DμOCT imaging permits the detailed resolution of the microarchitecture of the mammalian cochlear sensory epithelium—the organ of Corti—and can be used to indicate the metabolic status of the component sensory and non-sensory cells required for human hearing.

## 2 Materials and methods

### 2.1 DμOCT imaging system

**Figure 1:**
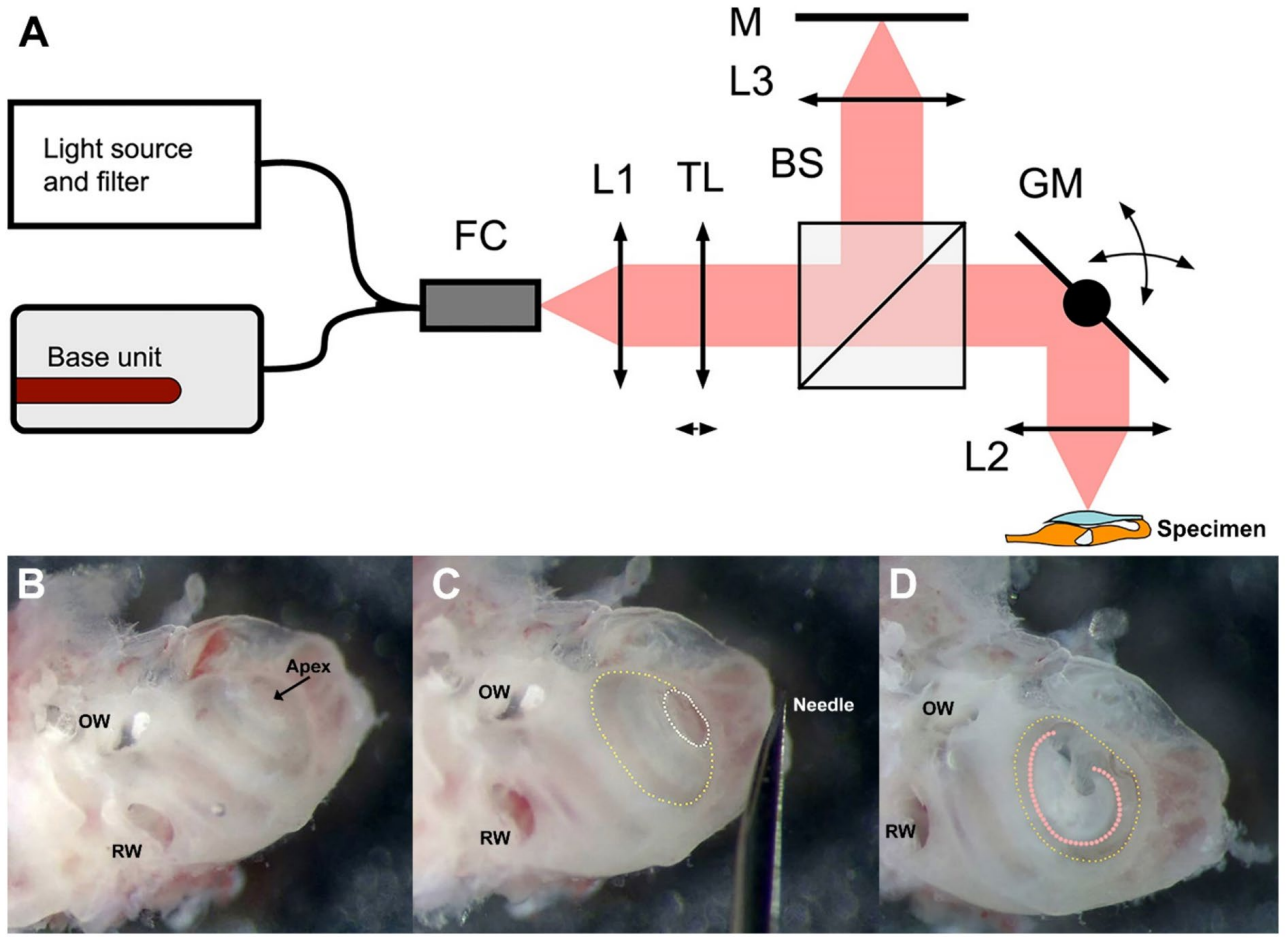
Schematic of the DμOCT benchtop imaging system **(A)** and preparation of the inner ear specimen for imaging **(B–D)**. **(A)** Simplified schematics of the DμOCT setup, with the organ of Corti within the mouse inner ear specimen displayed in the lower right-hand corner (not to scale). **(B–D)** Preparation and orientation of the adult mouse inner ear specimen for DμOCT imaging. The inner ear is positioned flat on the surface, resting on the inferior portion of the otic capsule and the anterior semicircular canal. Major anatomical landmarks including the RW and OW are identified prior to cochleostomy **(B)**. An initial cochleostomy is created in the cochlear apex (white dotted circle) and widened to the demarcation line (yellow dotted circle) of the apical to mid-basal turn region **(C)**. The inner ear is mounted on its anterior and posterior semicircular canals so the cochleostomy (yellow dotted circle) and the organ of Corti (orange dotted line) are facing upward for imaging, parallel to the bottom of the dish and surface of the medium **(D)**. BS, non-polarizing beam splitter; DμOCT, dynamic micro-optical coherence tomography; FC, 90:10 fiber coupler; GM, 2-axis galvanometer mirror scanning system; L1, collimator; L2, 0.3 NA microscope objective; L3, 0.3 NA microscope objective; M, mirror; NA, numerical aperture; OW, oval window; RW, round window; TL, liquid tunable lens.

Figure 1A depicts a schematic of the custom-built benchtop DμOCT imaging system. We utilized a modified Thorlabs Ganymede base unit (model GAN621-SP10, Thorlabs, United States) equipped with a supercontinuum source (SuperK Extreme EXR-15 OCT, NKT Photonics, Denmark). The light emitted by the supercontinuum source was filtered to a wavelength range of 600–1,000 nm and directed into a 90:10 fiber coupler (TW670R2A2, Thorlabs, United States). The fiber connector attached to the scan head was equipped with a fiber-based mirror tunnel that created multiple copies of the beam with different wavefronts through a self-imaging mechanism, enabling EDOF at the tissue (Yin et al., 2016; Yin et al., 2017). Ten percent of the light was passed through a reflective collimator (model RC08APC-P01, Thorlabs, US) into a customized modular OCT scan head (OCTP, Thorlabs, United States). This light passed through a tunable lens before being split into the reference and sample arms by a 30:70 non-polarizing beam splitter (BS). In the reference arm, light was transmitted through an objective lens (UMPLFLN 10XW Objective, Olympus, United States) and reflected back through the optical system from a mirror. Sample arm light was deflected by a pair of mirror-mounted scanning galvanometers that scan the light through an objective lens (UMPLFLN 10XW Objective, Olympus, United States) onto the exposed cochlea. The backscattered light from the cochlear tissue was sent back and recombined with the reflected reference light before it was coupled back into the fiber and sent to the spectrometer via the 90:10 fiber coupler. The remaining dispersion imbalance between the reference and sample arms was numerically compensated by optimization (Schulz-Hildebrandt et al., 2018a; Attendu et al., 2019). Equipped with a microscope objective with a NA of 0.3, the system captured μOCT images with a spatial resolution of at least 2 μm at a rate of 100,000 A-lines per second.

### 2.2 Preparation of mouse cochleae for imaging

A total of 8 adult wild-type CBA/CaJ mice (aged 6–16 weeks) were used in this study (*n* = 4 each of males and females). Six mice were used to develop the methodology for DμOCT imaging while two cochleae from two mice were used for the final imaging experiment presented in the figures. The development of the methodology included optimization of the inner ear extraction, specimen preparation and orientation, culture media, and non-toxic fixation to the dish, as well as tests to confirm resolution, focus depth, and other imaging parameters.

Mice were anesthetized with CO_2_ inhalation before sacrifice. After decapitation, the intact otic capsule (containing the cochlear membranous labyrinth of the inner ear) was extracted from the temporal bones with forceps and immediately immersed in culture medium consisting of 97% DMEM (#10313039), 1% N2 supplement (#17502048), 1% ampicillin in distilled water (#11593027), and 1% fetal bovine serum (#10437028, all ThermoFisher Scientific, United States). Under a dissecting microscope, a cochleostomy was created by carefully piercing the bone of the otic capsule with the needle of an insulin syringe (28 G, 329461, U-100 Insulin Syringe, BD, United States) and then sparingly widening the opening with #5 forceps (11251-10, FST, United States) to expose the organ of Corti of the apical turn and part of the basal turn (Figures 1B,C). Precautions were taken not to disturb the tectorial membrane and the sensory cells underneath. The otic capsule was mounted to the bottom of a 35 mm cell culture dish (#627160, Greiner Bio-One, United States) containing the aforementioned media using an inert non-toxic composite (Kwik-Sil adhesive, World Precision Instruments, United States). The cochlear apex was positioned up and parallel to the bottom of the dish, while the inner ear rested on the anterior and, partially, the posterior semicircular canals (Figure 1D).

### 2.3 DμOCT imaging

DμOCT imaging of morphological and metabolic features of the organ of Corti was conducted for approximately 150 frames through the cochleostomy described above, without further manipulation. The organ of Corti was identified for imaging based on its location relative to the otic capsule walls and using anatomical landmarks; first, the modiolus was located and, after zooming in, the tunnel of Corti, spiral limbus, and the tectorial membrane were used to orient to and focus on the sensory and supporting cells. Cross-sectional μOCT images were displayed in real-time. To determine whether DμOCT could detect metabolic changes in the cells of the organ of Corti, imaging was also conducted approximately 4 min after replacing culture media with 10% neutral buffered formalin.

### 2.4 Image reconstruction

The OCT fringe data were processed using standard OCT image reconstruction routines, including apodization, windowing, numerical dispersion correction, and Fourier transformation (Koprowski and Wróbel, 2011). Small global movements in axial directions between each time were corrected by calculating the average phase difference between subsequent A-lines along the time direction and multiplying the inverse phase difference to the following A-scan. Signal variation over time was analyzed by computing the frequency spectrum of the complex, motion-corrected dataset using a Fast Fourier transformation with 2 times zero-padding and assigning images in frequency bins to red-green-blue (RGB) images where blue (static) corresponded to tissue motion between 0–0.4 Hz, green (metabolic activity) to 0.4–5.1 Hz, and red (Brownian motion predominant) to 5.1–25 Hz (Leung et al., 2020; Münter et al., 2020). Each RGB channel was histogram-equalized based on the standard deviation (SD) of the time series (Kohlfaerber et al., 2022). When determining the SD of the complex, motion-corrected dataset, the dataset’s principal components were first calculated, followed by calculation of the SD of these principal components excluding the first principal component. Each individual channel underwent normalization by scaling between 0 and 1. The histogram of each normalized RGB channel was adjusted to increase morphological contrast. To accentuate cellular features in the image, we matched the histogram of each RGB channel to that of the SD image.

## 3 Results

**Figure 2:**
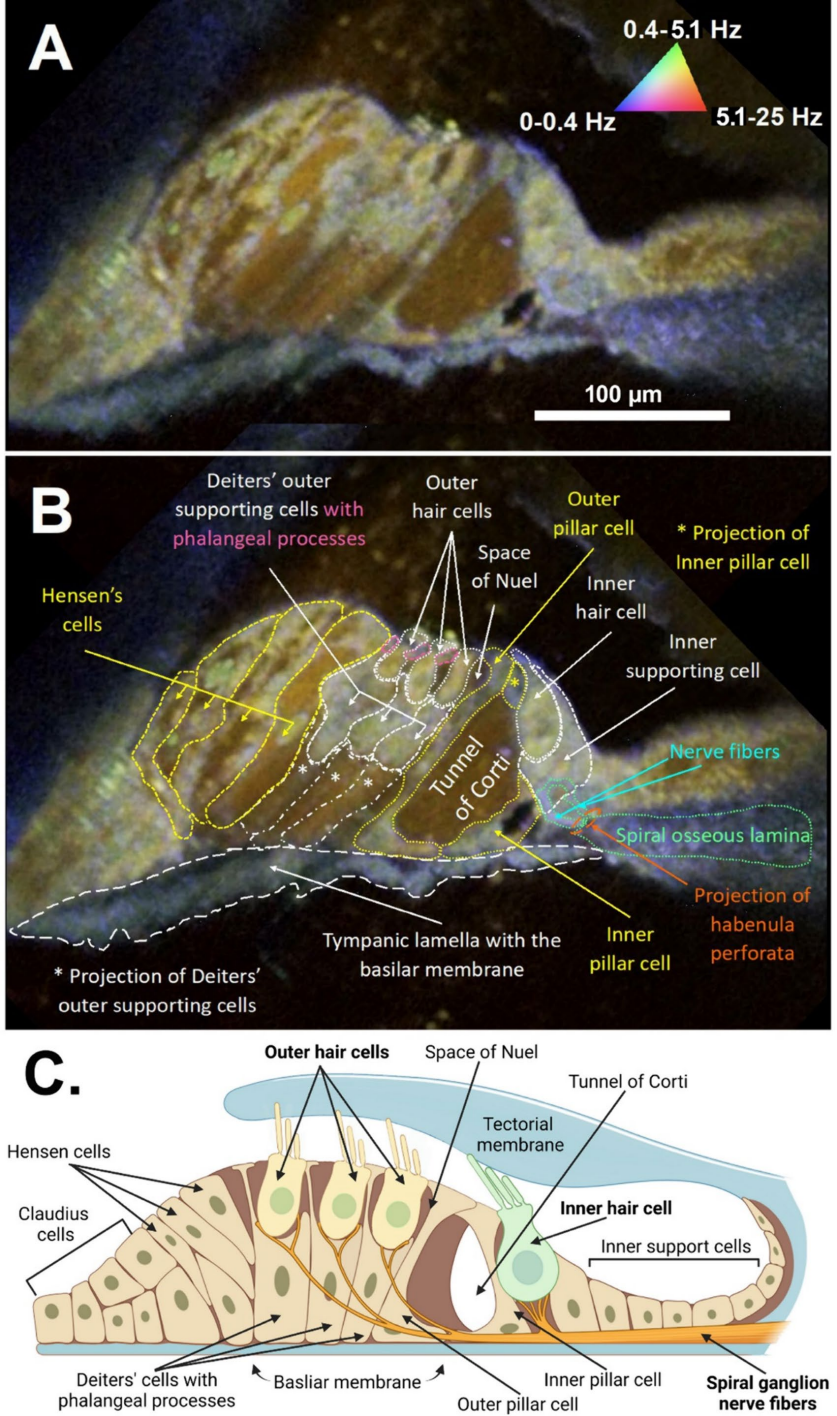
DμOCT cross-sectional image of the apical region of the adult mouse organ of Corti. DμOCT image of the adult mouse organ of Corti without **(A)** and with **(B)** anatomical features annotated. Static anatomical features (e.g., basilar membrane circled in white dashes) appear blue and Brownian motion-predominant areas, such as the tunnel of Corti, appear brown-red (triangle in **A**). Metabolically-active cell features appear green-yellow (nuclei, mitochondria, stereocilia). **(C)** Schematic of the organ of Corti, including the inner and outer hair cells and non-sensory cells (created with Biorender; www.biorender.com). DμOCT, dynamic micro-optical coherence tomography; Hz, hertz.

The anatomical and cellular features of the organ of Corti from a freshly excised adult mouse cochlea could be visualized in high detail using DμOCT imaging, permitting discernment of the boundaries of cells, fluid-filled spaces, and cell nuclei (Figure 2). Following image reconstruction, the resulting cross-sectional DμOCT image illustrated the static basilar membrane, reticular lamina, actin cytoarchitecture, and bone of the spiral osseus lamina in *blue* (0–0.4 Hz), while the fluid-filled tunnel of Corti, space of Nuel, and supporting (Hensen, Deiters’, and Claudius) cells’ cytoplasm appeared red/brown indicating Brownian motion (Figure 2B). Conversely, cell nuclei of supporting cells and hair cell stereocilia and cytoplasm appeared green-yellow in the image, indicating metabolic activity of their mitochondria (0.4–5.1 Hz). The mitochondria of inner hair cells (IHCs) and OHCs are distributed throughout the cells’ cytoplasm, comprising approximately 7–10 and <5% of their volume, respectively (Hashimoto and Kimura, 1988; Lysakowski et al., 2022). Both types of hair cells have higher mitochondrial density at the apical region near the cuticular plate to support mechanotransduction channels, as well as surrounding their nuclei (Fettiplace and Nam, 2019). However, OHCs also have higher density at the cell walls to support prestin-mediated motility while IHCs have more mitochondria in the basal region to support afferent synapses (Lysakowski et al., 2022).

**Figure 3:**
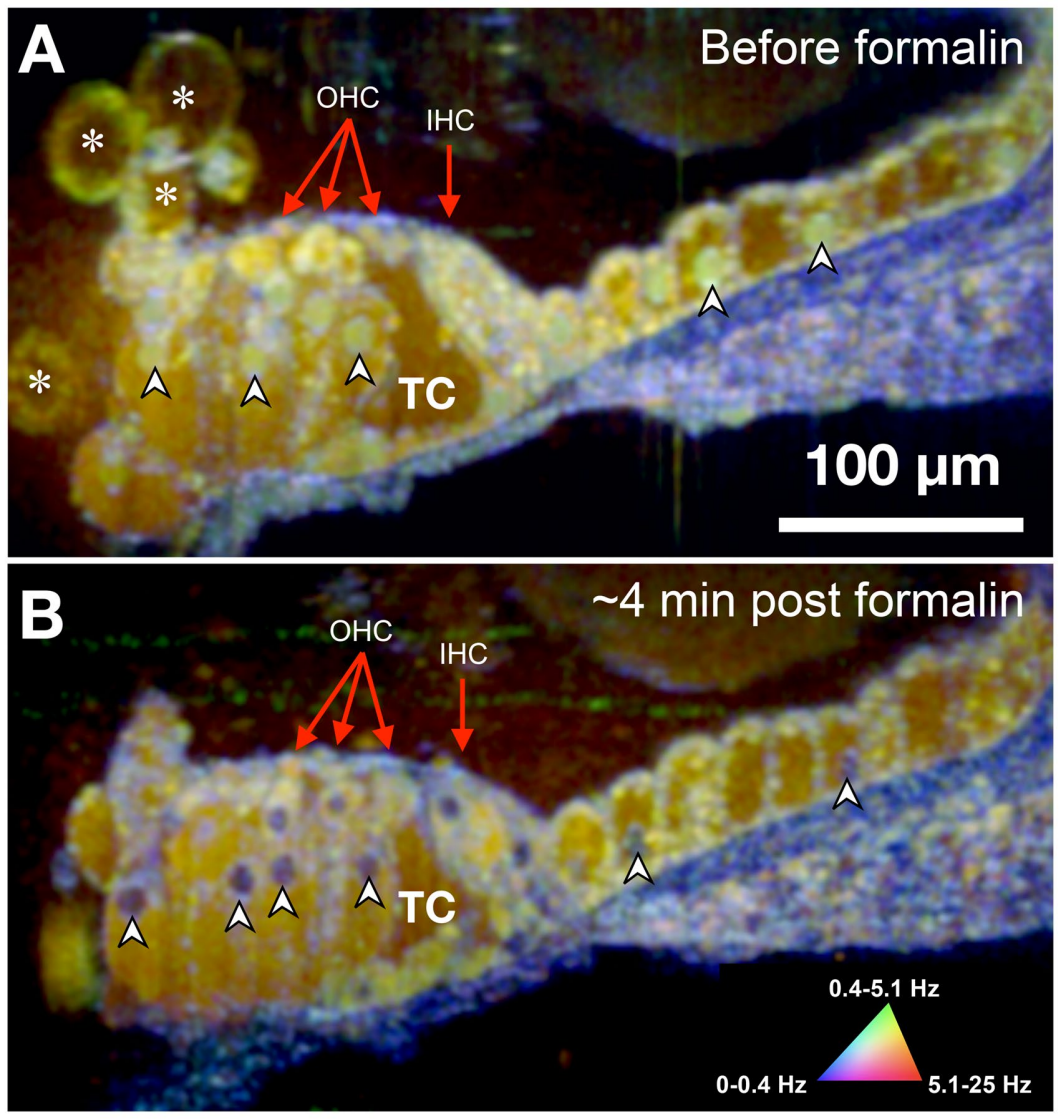
DμOCT image of adult mouse organ of Corti before **(A)** and after **(B)** formalin application. Images are an average of 5 consecutive DμOCT frames for improved clarity. Arrowheads point to examples of cell nuclei that shifted from active (green) to static (blue) post-formalin. Red arrows indicate the OHCs and IHCs. DμOCT, dynamic micro-optical coherence tomography; Hz, Hertz; IHC, inner hair cell; min, minutes; OHC, outer hair cell; TC, tunnel of Corti. * Indicates bubbles consistent with blebbing cells.

The sensorineural cells of the mammalian cochlea have high energy (ATP) needs and their precise metabolic functioning enables the perception of sound during hearing; conversely, alteration of the metabolic balance can be lethal for hair cells by disrupting mitochondrial activity required to sustain the intracellular ionic balance (O'Sullivan et al., 2023). Accordingly, we used formalin to test whether changes in the cell/tissue frequency content in response to cytotoxic stress alter the DμOCT signal characteristics indicating cell metabolism and viability. Formalin induces rapid protein–protein and DNA-protein crosslinking resulting in the arrest of cellular metabolism and other processes, but overall preservation of cell structure (Thavarajah et al., 2012). Figure 3 shows DμOCT images of a mouse organ of Corti after cochlear extraction and 4 min after formalin was applied to the culture media. Prior to formalin application to the sample, we observed numerous yellow-green cell nuclei and cytoplasmic contents indicating metabolic activity (Figure 3A), as well as the anatomical structures described in Figure 2 (i.e., sensory cells, supporting cells, and static actin microarchitecture or bone). However, within approximately 4 min after formalin application, nearly all cell nuclei appeared blue (static) (arrowheads in Figures 3A,B), presumably due to DNA-protein crosslinking, and the number of bright yellow-green organelle puncta had markedly declined. Additionally, the cytoplasmic contents of cells, particularly the hair cells, had begun to shift from bright yellow-green to muted yellow or brown indicating reduction in metabolic activity. The blue color of the static anatomical features of the organ of Corti, such as the basilar membrane, became more pronounced, as would be expected due to the crosslinking of actin or collagen proteins enhancing rigidity. Both samples shown in Figures 2, 3 were placed to allow the imaging beam to radially scan the organ of Corti in the apex region of the opened cochlea.

**Figure 4:**
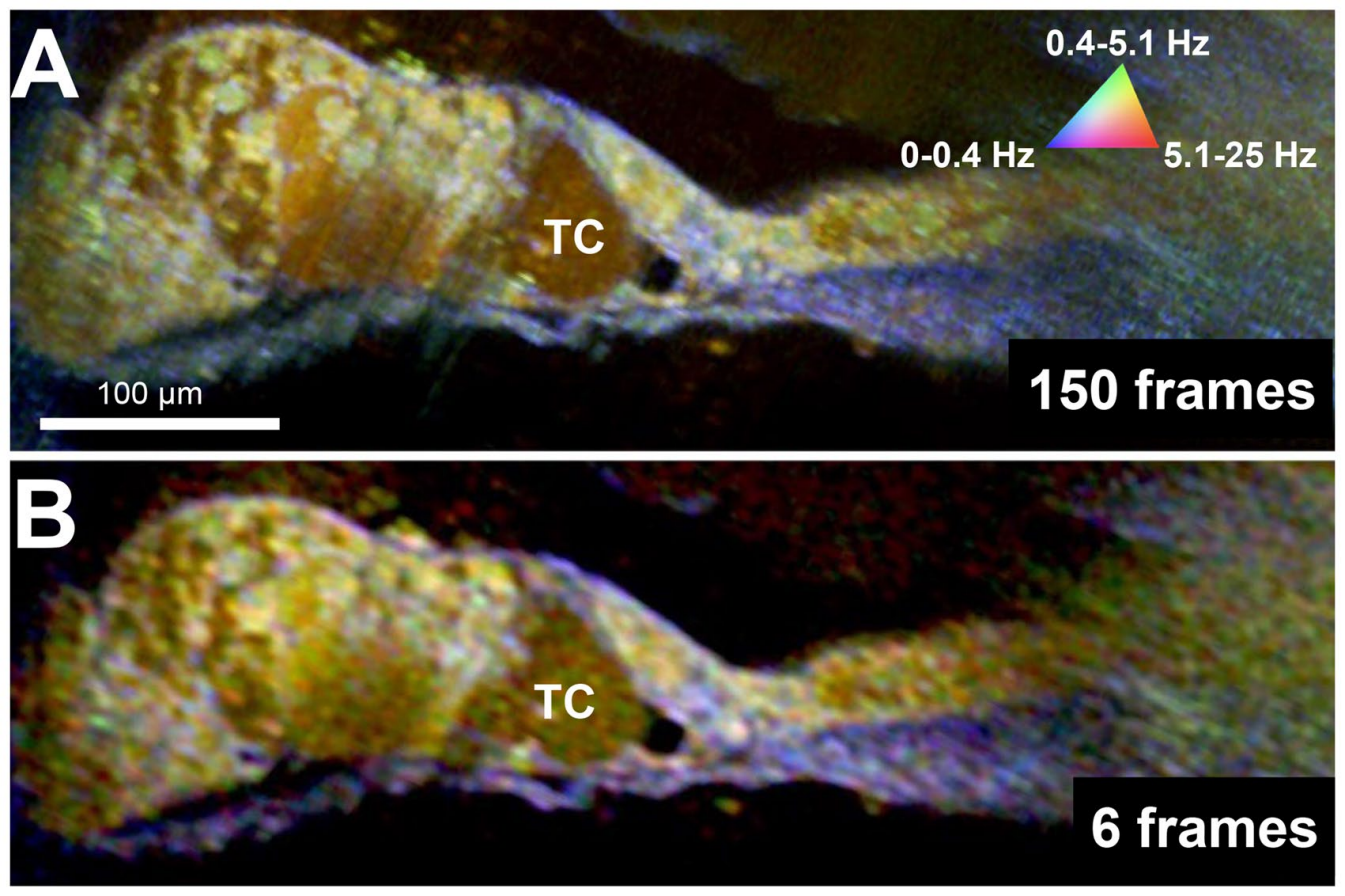
DμOCT images of adult mouse organ of Corti reconstructed from 150 contiguous frames over 1.5 s **(A)** and 6 contiguous frames spanning 375 ms **(B)**. DμOCT, dynamic micro-optical coherence tomography; Hz, Hertz; ms, milliseconds; s, seconds; TC, tunnel of Corti.

Because DμOCT requires multiple frames to be acquired from tissue at the same location, minimizing the number of frames needed to reconstruct an image—thereby reducing the frame rate, shortening the imaging time, and reducing the risk of damaging living tissue—is critical for determining the potential of DμOCT as a clinical endoscopic imaging tool. Upon testing the same image set from the cochlea shown in Figure 2, using the complex data combined with the standard variation-based contrast enhancement, we determined that as few as 6 frames, separated by 62.5 ms, can be used to reconstruct cochlear DμOCT images with sufficient detail to discern individual cells as well as their metabolic state (Figure 4). The reconstructed DμOCT imaging from 6 contiguous frames permits clear distinction of the cellular boundaries of the IHCs and OHCs, as well as the interface of the organ of Corti (yellow-green colored) and the basilar membrane (blue).

## 4 Discussion

Due to the myriad potential insults prompting SNHL, a detailed understanding of a patient’s unique inner ear pathology is essential to both develop and provide effective, personalized treatment. However, to date, there are no methods nor clinical tools which can assess cell morphology and function within the living human inner ear, hindering clinical evaluation, drug discovery, and treatment delivery. Here, we have presented DμOCT, an advancement in μOCT imaging which permits simultaneous assessment of the morphological details and metabolic state, via ATP-dependent intracellular motion, of the sensory and non-sensory cells of the organ of Corti *in situ*. DμOCT enabled the identification of individual cells and nuclei in as few as 6 frames and 375 ms and could capture rapid transitions in metabolic state following an insult.

This study is a descriptive demonstration of the potential of using DμOCT for high-resolution, metabolic activity-related functional imaging of the mammalian inner ear. As can be seen in Figures 2, 3, the two imaged cochleae have slight structural discrepancies (cell size, cell direction, etc.), primarily due to differences in tissue orientation and imaging location in the apical organ of Corti. However, the DμOCT frequency signatures for the various component cells appeared the same in both imaged cochleae and were also consistent across all other cochleae during the imaging protocol development. DμOCT surpasses standard OCT regarding its imaging resolution in both lateral and axial directions, providing a superior tool for imaging the human inner ear. Compared to our previous study using standard μOCT for inner ear imaging (Iyer et al., 2016), DμOCT substantially improved the intracellular contrast. Additionally, the change in DμOCT signature after formalin application to the cochlea demonstrates that DμOCT is capable of capturing metabolic activity with sub-cellular resolution.

Only one prior study has attempted to use dynamic OCT methods to collect structural and motion information from mammalian cochlear cells *ex vivo* (Serafino et al., 2024). Serafino et al. conducted dynamic OCT of mouse organ of Corti dissected from the cochlea and mounted in tissue culture slides. While larger anatomical features of the organ of Corti could be visualized using their instrumentation, degradation of lateral resolution prevented delineation of cell boundaries or the identification of cell organelles such as nuclei or mitochondrial distribution. In contrast, we conducted DμOCT imaging of the organ of Corti *in situ* (within the whole cochlea), using a window in the apical otic capsule for imaging access. Although the theoretical resolution of the OCT system in Serafino et al. was similar to ours (<2 μm), the current results demonstrate a high level of cellular and intracellular detail absent from the prior study’s images. The variance in tissue contrast between studies, particularly for “static” elements like bone, may be attributable to differences in instrumentation. Additionally, we demonstrate dynamic change in cochlear cell metabolism following an insult (formalin) as well as the ability to capture metabolic information very rapidly, in as few as 6 frames.

Despite the high prevalence of SNHL, our limited understanding of patients’ cochlear pathology has contributed to the longstanding failure to develop effective pharmaceutical, gene, or cell therapies toward restoring hearing (Jiam and Rauch, 2023). Although hearing aids and cochlear implants can sometimes improve hearing, these devices are not effective for patients lacking auditory neurons, cannot fully restore natural sound perception, and do not treat the underlying pathology. Clinical trials of candidate therapies have been attempted with the goal of prevention of SNHL (Kil et al., 2017) or prompting hair cell regeneration (Ren et al., 2019; Schilder et al., 2019; Le Prell, 2021; Li et al., 2024). Recently, gene therapy for otoferlin mutations is being tested in open-label clinical trials and early results illustrate the transformative potential of this approach (Lv et al., 2024). However, otoferlin gene therapy requires patients to have this rare mutation as well as normal cochlear microanatomy with intact, functional hair cells, limiting its applicability to a small patient population. Importantly, there have yet to be successful hair cell regeneration trials in patients with acquired SNHL, the most common type, despite multiple studies reporting promising evidence in animal models (Izumikawa et al., 2005; Hori et al., 2007; Kraft et al., 2013; Mizutari et al., 2013; Matsunaga and Nakagawa, 2023). Ultimately, the ability to screen for amenable cochlear phenotypes [i.e., hair cell functionality or absence of flat epithelium (Izumikawa et al., 2008; He et al., 2020)] with DμOCT could enable better patient selection and success of clinical trials by identifying who might benefit from a particular therapy. Further, the ability to image the sensorineural cells of the human cochlea *in vivo* and *in situ* could reduce the reliance on animal models and cadaveric specimens that may not reflect the true physiology or dynamics.

There remain several barriers to the clinical translation of DμOCT imaging for otology, including the challenges of accessing and imaging the cochlea’s spiraling organ of Corti within the temporal bone. The human cochlea is routinely accessed during surgical placement of cochlear implants (CI) that are used to treat severe-to-profound hearing loss. Minimally-invasive techniques are now the mainstay approach for CI (Labadie et al., 2014; Jiang et al., 2017), which can preserve patients’ residual hearing (Sprinzl et al., 2020). Previously, we have paired μOCT with flexible microendoscopy to image the human cochlea *ex vivo* (Iyer et al., 2021), implemented using self-imaging wavefront division pioneered in other forms of catheter-based μOCT (Yin et al., 2016; Yin et al., 2017; Yin et al., 2019). The μOCT microendoscope can be inserted into human cadaver scala tympani over the entire first 360-degree (basal) turn via the round window (a natural aperture) (Iyer et al., 2021), similar to the minimally-invasive surgical approach for CI electrode insertion. As an initial clinical application, DμOCT could visualize and enable the preservation of intracochlear cells or aid in optimal CI electrode placement, which are important for maximizing CI success (Nadol and Eddington, 2006; Pfingst et al., 2015).

An additional consideration for the clinical translation of DμOCT is that the cochlea is highly sensitive to mechanical, chemical, or thermal perturbation. The hair cells sit on the flexible basilar membrane and their projecting stereocilia are embedded in the tectorial membrane (Figure 2C). Thus, intense vibrations induce shearing forces which can trigger toxic influx of ions through the cells’ mechanotransduction channels, leading to cell apoptosis or dysfunction (Furness, 2015). Changes in perilymphatic pressure (i.e., due to leaks/fistula or elevated intracranial pressure) can also impair hair cell function and result in hearing loss (Heilen et al., 2020; Sarna et al., 2020). Further, heat itself is shown to activate the hair cell mechanotransduction apparatus (Azimzadeh et al., 2018), underscoring the need for rapid imaging when using laser light, such as we demonstrate in this article (Figure 4). Technical challenges for translation include the need for customized stabilization of the probe and compensation for biological sources of movement during image acquisition. We have developed and implemented software stabilization routines based on local elastic unwarping that are effective for compensating in-plane motion for DμOCT, as well as produced algorithms to adjust for biological sources of motion in μOCT images acquired from living animals and patients using endomicroscopic μOCT probes (Sorzano et al., 2005; Schulz-Hildebrandt et al., 2018b; Leung et al., 2019; Leung et al., 2020; Vijaykumar et al., 2023), which will facilitate motion stabilization *in vivo*.

DμOCT intracochlear imaging holds the potential to be the first high-resolution, cellular-functional imaging tool suitable for use in patients with SNHL, allowing real-time observation of metabolic and structural changes in specific cellular populations in the organ of Corti *in vivo*. Future studies exploring the potential clinical application of DμOCT, with or without paired endoscopy, could include imaging living tissue from freshly excised human temporal bones from organ donors (Vaisbuch et al., 2022) or of large animals *in vivo* to develop minimally-invasive techniques for probe insertion (Andres-Mateos et al., 2022).

## Data Availability

The original contributions presented in the study are included in the article/supplementary material, further inquiries can be directed to the corresponding authors.
